# Visual and anatomic outcomes of sustained single agent anti-VEGF treatment versus double anti-VEGF switching in the treatment of persistent diabetic macular edema

**DOI:** 10.1186/s40942-020-00220-5

**Published:** 2020-06-08

**Authors:** Osama A. Sorour, Keke Liu, Nihaal Mehta, Phillip Braun, Isaac Gendelman, Elsayed Nassar, Caroline R. Baumal, Andre J. Witkin, Jay S. Duker, Nadia K. Waheed

**Affiliations:** 1 New England Eye Center, Tufts Medical Center, Tufts University, Boston, MA USA; 2 Department of Ophthalmology, Tanta University, Tanta, Egypt; 3 University of Hawai’i John A. Burns School of Medicine, Honolulu, HI USA; 4 The Warren Alpert Medical School of Brown University, Providence, RI USA; 5 Yale School of Medicine, Yale University, New Haven, CT USA; 6 Tufts University School of Medicine, Tufts University, Boston, MA USA; 7 Department of Ophthalmology, Tufts Medical Center, 800 Washington Street, Box 450, Boston, MA 02111 USA

**Keywords:** Diabetic macular edema, Anti-VEGF, OCT, Diabetic retinopathy, Intravitreal

## Abstract

**Background:**

To compare the anatomical and visual outcomes in eyes with persistent diabetic macular edema (DME) after initial anti-VEGF therapy that were retreated continuously with the same anti-VEGF drug versus those that underwent two successive cycles of medication change in anti-VEGF drugs (double anti-VEGF switch).

**Methods:**

Retrospective review of eyes with persistent DME after 3 initial consecutive monthly anti-VEGF injections. This cohort was divided into two groups: Group 1 continued to receive the same initial anti-VEGF drug for at least 18 months while group 2 eyes were switched to different anti-VEGF medications twice. Group 1 was further subdivided into: Group 1A composed of eyes with less than 20% reduction in central subfield thickness (CRT) at month 3; and group 1B eyes with greater than or equal to 20% reduction in CRT. The percentage of eyes that achieved greater than 10 letters visual acuity (VA) gain or loss was recorded as the primary end point (through month 18 in group 1 and month 6 after 2nd switch in group 2).

**Results:**

Group 1A, 1B and group 2 were composed of 24, 18, and 14 eyes respectively. 34.7%, 56.2% and 36.3% of eyes achieved > 10 letters gain, while 4.3%, 6.2% and 27.2% of eyes lost > 10 letters in groups 1A, 1B, and 2, respectively. Analysis of the visual acuity (VA) letter change in this time interval revealed no significant difference between all groups (p = 0.11). Mean VA and CRT measurements at the primary endpoint in all groups were 0.5, 0.39, and 0.47 logMAR (p = 0.44), and 369.7, 279.9, 321 µm, (p = 0.01) respectively.

**Conclusions:**

There was no difference in the visual outcomes between the two treatment strategies in eyes with persistent DME after 3 consecutive anti-VEGF injections. This may indicate that anti-VEGF switching—even if it is done twice—may have comparable clinical outcomes to sustained treatment with one agent.

## Background

The worldwide prevalence of diabetes has progressively increased in recent decades and is predicted to grow to 430 million by 2030 [1]. Diabetic retinopathy (DR) is a common sequelae for diabetic patients, and represents a major cause of acquired blindness worldwide, with proliferative diabetic retinopathy (PDR) and diabetic macular edema (DME) as the main causes of visual deterioration in DR patients [2]. Laser photocoagulation was the standard treatment for DME until clinical trials demonstrated that intravitreal injection of pharmacological agents (steroids and anti-VEGF agents) can achieve significant anatomical and visual improvement in DME patients with less adverse effects. Consequently, anti-VEGF injection has become first-line treatment for eyes with center-involving diabetic macular edema (CI-DME) and worsening visual acuity based on findings from DCRC.net and pivotal studies like RISE/RIDE, HARBOR and Protocol V [3, 4]. However, despite the intensive intravitreal injection schedules used in clinical trials, some eyes still have residual DME. Between 30 and 65% of eyes fail to achieve complete resolution of retinal thickening after 1 to 2 years of treatment [5–7]. Although there is a lack of consistent nomenclature for DME that does not fully resolve following initial anti-VEGF treatment, it is usually referred to as persistent diabetic macular edema (PDME) [8].

Various studies have demonstrated long-term visual acuity loss for patients with even minimal prolonged persistent retinal edema. Patients in the RISE/RIDE trials who were initially in the laser group and then switched to receive ranibizumab after month 24 failed to achieve similar visual gains compared with patients that received ranibizumab at the baseline of the trials, indicating the importance of achieving early macular dryness to protect the retinal photoreceptors and maintain full vision improvement potential [9].

The choice of initial anti-VEGF agent for DME treatment is based on several factors including availability, efficacy and cost. When a patient does not respond to the initial agent after several monthly injections, many ophthalmologists switch to another anti-VEGF agent, especially if the initial treatment agent was bevacizumab. There have been several studies demonstrating functional and anatomical benefits of switching anti-VEGF agents in PDME treatment, although there are no consistent switching rules across studies [10]. However, reports from the BOLT, RISE/RIDE, and DRCR.net studies have demonstrated that DR patients with DME that did not achieve immediate anatomical response may be late responders, as they still achieved further functional and anatomical improvement with sustained treatment [10–13]. This finding makes unclear whether the visual and/or anatomic improvement seen in patients that switch anti-VEGF therapies originates from the new intravitreal anti-VEGF agent or from the total number of anti-VEGF injections [14]. The scenario becomes more complicated when the patient did not respond adequately after switching anti-VEGF; in this situation, it is unclear whether a second switch would be more beneficial versus continuing the same treatment regimen. This study investigates the visual and anatomical outcomes after double switching of anti-VEGF agent versus sustained treatment with the same drug.

## Methods

This was retrospective, observational, comparative case-series study. The protocol of this study was approved by the Institutional Review Board at the Tufts Medical Center. The research adhered to the Declaration of Helsinki and the Health Insurance Portability and Accountability Act.

The electronic health records of patients who received anti-VEGF treatment for center-involving DME by the Retina Service at the New England Eye Center (NEEC) between January 2010 and December 2018 were retrospectively reviewed to identify potential study participants. Inclusion criteria comprised type 1 or type 2 diabetic patients aged ≥ 18 years with best corrected visual acuity (BCVA) ≥ 20/400 in the study eye. Center-involved DME was defined as central retinal thickness (CRT) ≥ 300 μm by Cirrus OCT (Carl Zeiss Meditec, Dublin, CA, USA) at the beginning of the study [4, 15]. Diabetic eyes with evidence of residual intraretinal and/or subretinal fluid and CRT on OCT > 300 μm after receiving loading doses of 3 monthly intravitreal anti-VEGF injections were considered to have PDME [16, 17]. The final cohort included eyes that either continued the same initial anti-VEGF drug after they met previous criteria for PDME as group 1, and eyes with two successive cycles of switching anti-VEGF drugs (double anti-VEGF switch) as group 2. Group 1 was further subdivided into eyes which demonstrated a decrease in CRT < 20% after three anti-VEGF injections (subgroup 1A), and eyes that achieved decrease in CRT ≥ 20% at month 3 (subgroup 1B). Patients in group 2 received at least three consecutive anti-VEGF injections, then switched to another anti-VEGF drug for at least 3 consecutive injections. After still showing PDME, they were again switched for a second time, which henceforth will be referred to as the 2nd switch.

Patients were excluded if they had any of the following treatments within 6 months prior to study entry: intravitreal or sub-tenon steroid, macular laser photocoagulation, panretinal photocoagulation, cataract surgery or pars plana vitrectomy. Patients who had vitreoretinal interface disorders, macular edema secondary to a cause other than diabetes or any concomitant ocular pathologies other than diabetic retinopathy were excluded. Patients with previous anti-VEGF treatment less than 6 months before the study were not included. This study included only eyes that received their successive anti-VEGF injection less than 12 weeks apart.

Data collected from chart review included medical history, type and date of intravitreal injections, slit-lamp biomicroscopy, intraocular pressure, color fundus photographs, fundus fluorescein angiography if available, BCVA and CRT. Central retinal thickness (CRT) was defined as the mean thickness of the neurosensory retina in the central 1 mm diameter subfield, computed with the OCT mapping on the Cirrus OCT (Carl Zeiss Meditec, Dublin, CA, USA) by trained OCT technicians, with registered repeat scans to prior reference scan. Visual acuity was converted to logarithm of minimum angle of resolution (logMAR) for statistical analysis.

The primary outcome was the percentage of eyes that achieved more than 10 letters gain and more than 10 letters loss in VA at the endpoint, which was specified as month 18 for group 1 and month 6 after 2nd switch for group 2. These outcomes were determined in comparison both to baseline visual acuity and to the transition point, which was defined as month 6 from beginning of treatment in group 1, and at time of 2nd switch in group 2. This measurement from the transition point was selected to analyze and compare the effect of sustained treatment after resistance to treatment through month 6 in group 1 and the effect of 2nd switch only in group 2 with subtraction of the initial effect of loading dose and 1st switch. Secondary outcome measurements included change in VA and CRT measurements throughout the study.

Statistical analysis was performed using SPSS v25 (IBM Corp, Armonk, NY, USA). Non-parametric tests were used as the distribution of the variables measured using the Kolmogorov–Smirnov test and Shapiro–Wilk revealed non-normal distribution among most variables. The Kruskal–Wallis and Mann–Whitney U test were used for the analysis of differences in VA and CRT measurements at the primary endpoints between groups and for analysis of the differences in degree of change in visual acuity at the primary endpoint in comparison to baseline and transition points. The Wilcoxon signed-ranks test was used for the analysis of VA and CRT measurements change within each group.

## Results

Fifty six diabetic eyes (n = 56) met inclusion criteria composed of group 1 (n = 42) eyes with PDME that continued same initial anti-VEGF agent and group 2 (n = 14) eyes with PDME and double anti-VEGF switch. The third anti-VEGF agent in group 2 was different in 8 eyes and the same as the first agent in 6 eyes. Group 1A and 1B included 24 and 18 eyes, respectively. Each group contained one eye per subject. Baseline demographics are presented in Table 1.

**Table 1:** Baseline characteristics of the study patients

	Group 1		Group 2	p-value
	Subgroup 1A	Subgroup 1B		
Age, mean (SD)	65.5 ± (10.4)	63.6 ± (7.1)	72.5 ± (8.1)	0.005**
Sex				
Female	13 (54.2%)	6 (33.3%)	5 (35.7%)	0.267
Male	11 (45.8%)	12 (66.7%)	9 (64.3%)	
Type of DM				
Type 1	5 (20.9%)	4 (22.2%)	2 (14.3%)	0.82
Type 2	19 (79.1%)	14 (77.8%)	12 (85.7%)	
Last known HBA1C, mean (SD)	7.2 ± (0.8)	7.5 ± (1.5)	7.5 ± (1.2)	0.91
Insulin use (%)	17 (70.8%)	12 (66.7%)	8 (57.1%)	0.89
Anti-VEGF treatment Naïve eyes (%)	14 (58.3%)	9 (50%)	9 (64.2%)	0.71
Prior focal laser treatment (%)	10 (41.6%)	6 (33.3%)	7 (50%)	0.64
Prior PRP treatment (%)	5 (20.8%)	2 (11.1%)	0 (0%)	0.17
Baseline logMAR VA, mean (SD)	0.58 ± (0.29)	0.65 ± (0.34)	0.55 ± (0.24)	0.87
Baseline CRT measurement (µm) (SD)	445.92 ± (126.4)	537.72 ± (131.7)	410.87 ± (86.4)	0.011*

*PRP* panretinal photocoagulation, *CRT* central retinal subfield thickness

*Group 1B patients had significantly thicker baseline CRT than other two groups. All other baseline demogrpgic data were not different between all groups

**Group 2 patients had significantly older age than other groups

There was no significant difference in sex distribution, type of DM, last known HbA1c, use of insulin, the distribution of eyes that received focal laser treatment, or panretinal photocoagulation prior to enrollment between all groups. However, patients in group 2 demonstrated a significantly older age than other groups (Table 1). 58.3%, 50.0% and 68.4% of eyes in groups 1A, 1B, and 2, respectively, were anti-VEGF-naïve at the point of enrollment in the study. Patients in group 1 received continued treatment for at least 18 months and follow-up to 24 months was available in 32 eyes (76%) with comparable results. The intravitreal drugs used for treatment were aflibercept, ranibizumab, or bevacizumab in 9, 9, and 6 eyes, respectively, in group 1A; and in 7, 8, and 3 eyes, respectively, in group 1B (Table 2). Patients received an injection every 6 weeks on average, with a mean total number of injections of 11.8 and 13.1 in group 1A and 1B, respectively. In group 2, patients received an average of 4.9 injections before the 1st switch, 6.9 injections between the 1st and 2nd switch, and 3.8 injections after 2nd switch (Table 2). Eight eyes in this group received bevacizumab, then switched to ranibizumab, and finally switched to aflibercept. In the other 6 eyes, bevacizumab was the initial drug in four eyes, then 2 eyes received aflibercept and the other 2 eyes received ranibizumab before the all 4 eyes received bevacizumab for the 2nd switch. The last 2 eyes in group 2 were treated initially with ranibizumab, then aflibercept, then ranibizumab. All patients in groups 1 and group 2 were treated regularly at less than 12-week intervals between successive injections.

**Table 2:** Injection *c*haracteristics in study groups

	Group 1 (42 eyes)			Group 2 (14 eyes)		
		Subgroup 1A	Subgroup 1B	Baseline to 1st switch	1st switch to 2nd switch	After 2nd switch
Type of treatment	Avastin	6 (25%)	3 (16.7%)			
	Lucentis	9 (37.5%)	8 (44.4%)			
	Eylea	9 (37.5%)	7 (38.9%)			
		p = 0.86				
Duration of treatment	18 months	42 eyes (100%)		34.3 ± (16.1)	50.8 ± (35)	60.2 ± (31.4)
	24 months	32 eyes (76%)				
Number of injections, mean (SD)		11.8 ± (3.2)	13.1 ± (3.1)	4.9 ± (2)	6.9 ± (4)	3.8 ± (1)
Interval between injections, mean (SD)		1.8 ± (0.2)	1.7 ± (0.3)	1.6 ± (0.3)	1.7 ± (0.4)	1.6 ± (0.3)

### Primary outcome measures

**Fig. 1:**
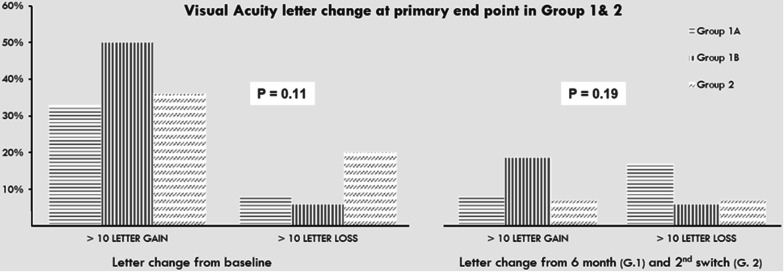
Analysis of change in visual acuity through the primary end point between all groups. A diagram illustrating the difference between groups 1A, 1B, and 2 in the amount of visual acuity (VA) letters change at the primary endpoint in comparison to VA at baseline (left), and to transition point (right, P=). There was no significant difference between all groups in both time intervals

Mean baseline visual acuity measurements were 0.58 ± 0.29, 0.65 ± 0.34, and 0.55 ± 0.24 logMAR units in groups 1A, 1B, and 2, respectively, with no significant difference in baseline measurement between all groups. At the primary end point of the study, VA measurements were 0.5 ± 0.3, 0.39 ± 0.2, 0.47 ± 0.3 logMAR, (p = 0.44); the percentage of eyes that achieved more than 10 letters gain from baseline VA was 34.7%, 56.2%, and 36.3%, and the percentage of eyes that lost more than 10 letters were 4.3%, 6.2%, and 27.2% in groups 1A, 1B, and 2, respectively. In comparison to VA measurements at the transition point (month 6 in group 1 and at 2nd switch in group 2), 21.7%, 18.7%, and 7.1% of eyes achieved > 10 letters gain, while 17.3%, 6.25%, and 7.1% of eyes demonstrated > 10 letters loss in groups 1A, 1B, and 2, respectively. There were no significant differences between all groups in the change of VA at the primary endpoint compared to both baseline and transition points (p = 0.11 and p = 0.9 respectively) (Fig. 1).

### Secondary outcome measures

**Fig. 2:**
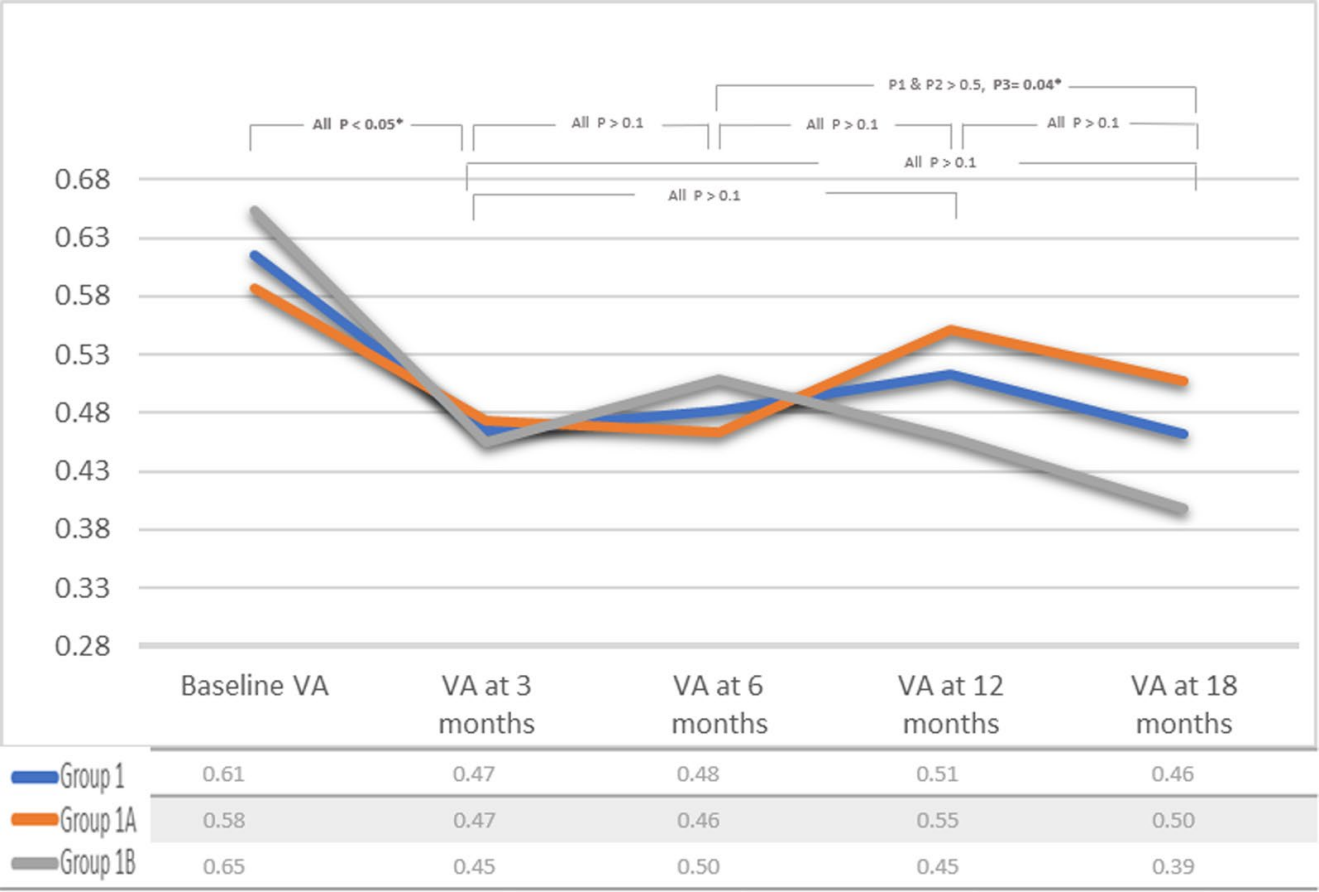
Mean visual acuity measurements in group 1 throughout study. A chart illustrating mean logMAR visual acuity in group1 and subgroups 1A and 1B through 18 months of sustained same agent anti-VEGF treatment. Data were collected at baseline, and after 3, 6, 12 and 18 months. P1 = p value in group 1, P2 = p value in subgroup 1A, P3 = p value in subgroup 1B

**Fig. 3:**
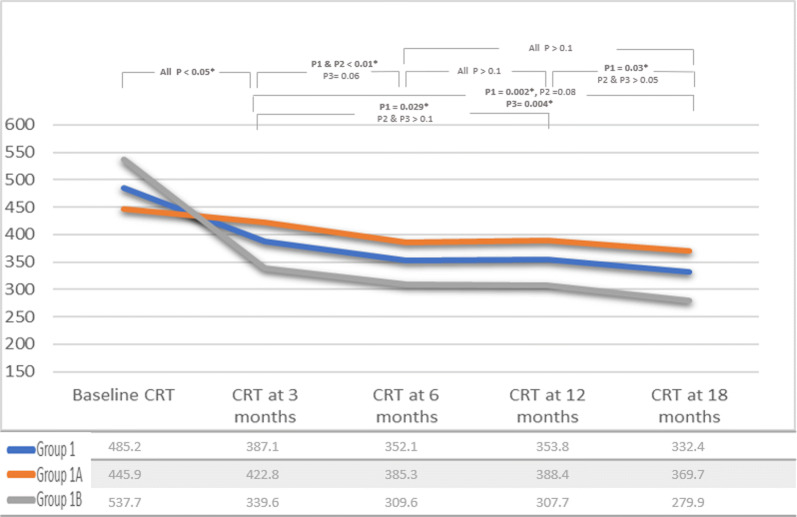
Mean CRT measurements in group 1 throughout study. A chart illustrating mean central retinal thickness (CRT) in group 1 and subgroups 1A and 1B through 18 months of sustained same agent anti-VEGF treatment. P1 = p value in group 1, P2 = p value in subgroup 1A, P3 = p value in subgroup 1B

In group 1A; VA and CRT measurements were 0.58 ± (0.29), 0.47 ± (0.28), 0.46 ± (0.3), 0.55 ± (0.37), 0.50 ± (0.3) logMAR, and 445.9 ± (126.4), 422.8 ± (112.7), 385.3 ± (111.4), 388.4 ± (106.4), 369.7 ± (120.3) µm at baseline, month 3, 6, 12 and 18, respectively (Figs. 2, 3).

In group 1B, VA and CRT measurements were 0.65 ± (0.34), 0.45 ± (0.31), 0.50 ± (0.38), 0.45 ± (0.31), 0.39 ± (0.29) logMAR, and 537.7 ± (131.7), 339.6 ± (86.9), 309.6 ± (52.7), 307.7 ± (71.3), 279.9 ± (67.5) µm at baseline, month 3, 6, 12 and 18, respectively (Figs. 2, 3).

**Fig. 4:**
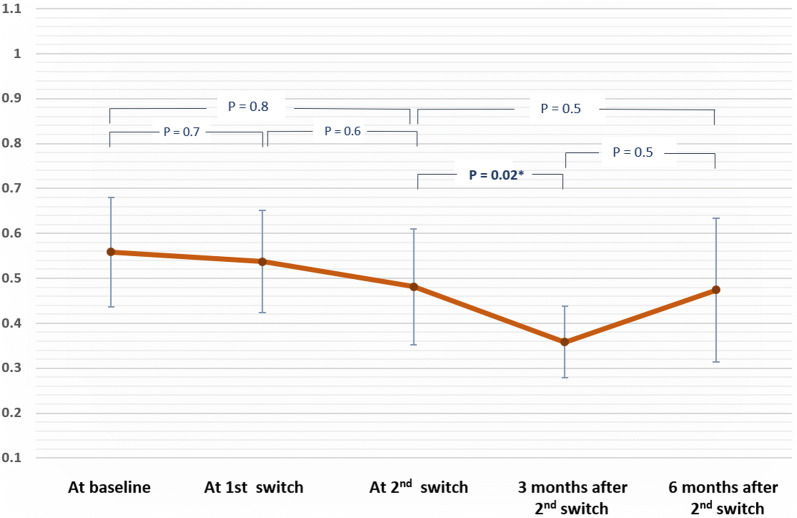
Mean visual acuity measurements in group 2 throughout study. Line chart illustrating the logMAR visual acuity in group 2 throughout the study period. All comparisons revealed insignificant changes. Significant improvement in VA was found shortly after 2nd switch before it was rapidly lost

**Fig. 5:**
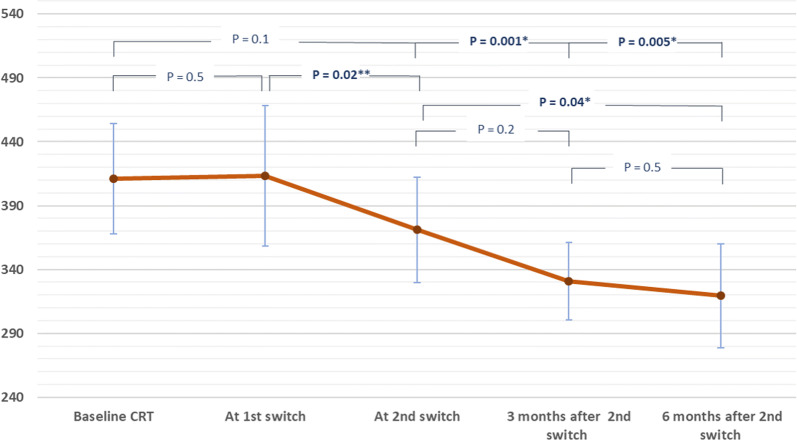
Mean CRT measurements in group 2 throughout study. Line chart illustrating the mean central retinal thickness in group 2 throughout the study period. Significant improvement was found after the 1st switch and at month 6 after the 2nd switch in comparison to the time of 2nd switching. Significant reduction of CRT was achieved after 1st switch in comparison to baseline and was maintained at month 3 and month 5 after the 2nd switch

In group 2, VA and CRT measurements were 0.55 ± (0.24), 0.53 ± (0.22), 0.48 ± (0.25), 0.35 ± (0.15), 0.47 ± (0.31) logMAR, and 410.8 ± (86.4), 413.4 ± (110), 371.0 ± (82.4), 330.9 ± (60.6), 321 ± (81) µm at baseline, time of 1st switch, time of 2nd switch, 3 and 6 months after 2nd switch, respectively (Figs. 4, 5). Analysis of CRT measurements at the primary end point revealed that eyes in group 1B were significantly thinner than those in group 1A (p = 0.03), but not statistically different than those in group 2 (p = 0.28, overall p between 3 groups = 0.01).

Analysis of VA in group 2 after the specified end point (n = 11) revealed significant improvement at month 18 compared to baseline and at the time of the 2nd switch (p = 0.04 and 0.38, respectively). However, no significant change in VA was found between groups 1A, 1B, and 2 (p = 0.27).

## Discussion

Although intravitreal anti-VEGF injections are the standard treatment for CI-DME, a significant percentage of patients initially show incomplete anatomic and/or visual response to treatment. An analysis of the DRCR.net protocol T study revealed that the incidence of persistent DME after 3 consecutive monthly injections was 50.8%, 53.2% and 72.9% through week 12, and 31.6%, 41.5% and 65.6% through week 24 in eyes that received aflibercept, ranibizumab and bevacizumab, respectively. Different treatment strategies have been proposed for PDME including switching to a different anti-VEGF agent, changing to intravitreal corticosteroid, focal laser treatment, and use of other adjunctive treatments like anti-oxidants [15, 16, 18, 19]. There is increasing data in the literature that show continued treatment with the same anti-VEGF drug may lead to both functional and anatomical improvement in the longer term [10]. However, nearly all of this data comes from post hoc analysis of clinical trials, and there is a paucity of studies specifically designed to answer this question. In our study, we have compared the short-term visual and anatomical outcomes of 18-month sustained treatment with one anti-VEGF drug (group 1) to double anti-VEGF switch (group 2) in the treatment of eyes with PDME.

In group 1 of our study, visual acuity measurements showed significant improvement at month 3 compared to baseline. After this initial improvement, VA demonstrated a temporary plateau, and no difference was noted between step intervals at month 6 and month 12. Moreover, in group 1A, mean VA slightly deteriorated between month 6 and month 12, leading to loss of significant difference of VA measurements in comparison to baseline VA. Nevertheless, vision improved again in both group 1A and 1B—a statistically significant improvement in VA in group 1B was found at month 18 compared to month 6 (Fig. 2). For CRT, there was a significant decrease at month 3 compared to baseline, at month 6 compared to month 3, and a further but non-significant decrease was found at month 12 compared to month 6. Further statistically significant decrease of CRT was present in month 18 as compared to month 12 (Fig. 3). In our cohort, patients with PDME through month 6 but with initial decrease of CRT ≥ 20% were more likely to achieve significant visual gain through month 18 of continuing treatment with same anti-VEGF drug. Patients with initial decrease of CRT < 20% were more susceptible to fluctuations in VA through the course of treatment with the same anti-VEGF drug, although the final visual outcome was the same in both groups.

Our results are comparable to previous studies. For example, Dugel et al. demonstrated that eyes with early limited anatomical response (< 20% decrease in CRT) to ranibizumab were significantly less likely to achieve > 20% reduction in CRT at 1 and 3 years of treatment compared to eyes with an early strong anatomical response which were able to maintain this response. However, there was no difference in long-term improvement in visual acuity after controlling for other confounders. This affirms the absence of association between OCT-derived early anatomical response and long-term BCVA improvement [20].

In a post hoc analysis of data from RISE/RIDE, patients that achieved > 10% decrease in CRT after 3 months (immediate responders) were compared to those with < 10% decrease in CRT (delayed responders). At month 24, delayed responders had thicker retinas and less CRT reduction from baseline compared with immediate responders, while both groups of patients had comparable BCVA gains and DR severity grade improvement from baseline [12]. Similar results were obtained from Koyanagi et al., who retrospectively evaluated patients that continued the same anti-VEGF treatment for 12 months. Patients were divided into two groups after 3 anti-VEGF injections based on improvement in the BCVA (visual improvement group and visual non-improvement) and CRT (delayed responder group with < 25% decrease in CRT, and immediate responder group with > 25% decrease in CRT). Using the anatomical response-based classification, both groups had comparable visual improvement at month 12 (p = 0.75), with a statistically significant reduction in CRT in the immediate responder group (p = 0.01). However, using a visual response-based classification demonstrated no difference in the anatomical outcome between both groups at month 12 [21].

In group 2 of our study, no significant change in VA was found after the 1st switch. However, at month 3 after the 2nd switch, there was a significant improvement in VA. This improvement faded gradually, and at month 6 no significant change was found compared to VA at 2nd switch (Fig. 4). Decrease in CRT with anti-VEGF switching was more pronounced than VA improvement, with significant reduction in CRT found after the 1st switch and 6 months after the 2nd switch, suggesting added visual and anatomical benefit of double anti-VEGF switching in patients with PDME (Fig. 5). To our knowledge no previous study has investigated the outcome after two successive cycles of anti-VEGF switching.

Our results are comparable to the previous literature of improved visual acuity and reduced CRT with anti-VEGF switching. Rahimy et al. retrospectively evaluated 50 eyes with PDME that converted to receive at least two aflibercept injections after at least four consecutive ranibizumab/bevacizumab injections. The mean BCVA improved from 0.60 to 0.55 logMAR (p = 0.12), and the average CRT decreased from 459.2 to 348.7 µm (p < 0.0001) by the second follow-up visit. However, in the subgroup of patients that completed 4 post-switch visits (44% of eyes), there was a significant improvement in vision (change from 0.8 to 0.65 logMAR, p = 0.003), suggesting that visual improvement may lag behind apparent resolution of fluid in DME [22]. Similar results of consistent reduction in CRT and variable VA improvement after anti-VEGF switch were found in other studies [23–25]. Other previous studies have also demonstrated that visual recovery does not parallel the anatomical improvement in DME treatment, with only 18% to 45% of patients showing BCVA gain ≥ 15 letters after 2 years of treatment. Several theories about this functional impairment have been suggested, including microstructural defects in the photoreceptors and external limiting membrane occurring in the fovea after a DME episode, neural apoptosis, glial reactivity, malfunction due to ischemia, and/or reduction in the thickness of the inner retinal layers [26].

In our study, the comparison of visual outcome between group 1 with 18-month sustained anti-VEGF treatment versus group 2 with double anti-VEGF switch revealed no significant difference with regards to BCVA letter change at the primary end point. In addition, final VA measurements were the same in all groups. However, final CRT measurements revealed significantly thinner retinae in patients with sustained anti-VEGF treatment with initial decrease of CRT ≥ 20% than in those with < 20% initial decrease in CRT. Our results agree partially to a small comparative study of patients with CI- DME ≥ 350 μm who continue ranibizumab injections versus those who converted to aflibercept. In both groups, there was significant anatomical improvement, while the decrease in CRT in the switch group was significantly more pronounced. On the other hand, functional improvement was not significantly different either between the baseline and last visits in both groups or between each other [14]. However, this study did not include eyes with double anti-VEGF switch. Interestingly, the American Society of Retina Specialists (ASRS) preferences and trends (PAT) survey in 2018 demonstrated that 69.8% of US practitioners would switch anti-VEGF agents when faced with a case of unresponsive DME, 7.7% would switch to steroids alone, 19.9% would incorporate steroids in combination with an anti-VEGF agent, and only 2.6% would choose another treatment. In case of pseudophakic patients, these trends become 56.9%, 15.6%, 26.5%, and 1.1%, respectively [27]. According to our study’s results, staying on course with the same agent would be a comparable alternative strategy to anti-VEGF switching.

Limitations to this study include its retrospective chart review nature. Only a small number of patients were eligible, which did not allow for analysis of all potential cofactors like type of used anti-VEGF or number of injections and most importantly, coexisting macular ischemia. Patients in group 2 were older than other groups, and pre-enrollment CRT was thicker in group 1B. These differences may present bias. Other limitations included the difference in primary endpoint between both groups; however, this discrepancy was attributed to the different nature of both groups and the aim of this study. As we are investigating the effect of sustained treatment in group 1 and studying the possibility of late response in these initially treatment-resistant patients, this analysis needed the primary end point to be as far as possible. In the switch group, the primary end point needed to be at a reasonable interval after the switch to analyze its effect. If we used similar primary end point at 18 months after 2nd switch—like group 1—the analyzed effect would be a combination of switch and sustained treatment, not the switch effect only.

## Conclusions

Diabetic eyes with PDME after a loading dose of 3 monthly anti-VEGF injections who continued on treatment with the same anti-VEGF demonstrated anatomical improvement; however, visual gains were not significant except after the initial 3 months of initiation of treatment. Eyes with double anti-VEGF switch demonstrated significant anatomical improvement after the first switch and 6 months after the second switch, and only short-term visual improvement after the second switch. No difference in visual benefit was found between both treatment strategies, with slight risk of visual loss when using either treatment method, suggesting that maintaining treatment using the same agent may be an appropriate strategy in the management of PDME. However, further prospective randomized studies with larger populations of patients, and avoidance of sources of bias are needed to elaborate final decisive conclusions.

## Data Availability

The data that support the findings of this study are available from Tufts Medical center. Data are available from the authors upon reasonable request and after permission of Tufts Medical Center.

## Abbreviations

Anti-VEGF: Anti-vascular endothelial growth factor
BCVA: Best corrected visual acuity
CRT: Central retinal subfield thickness
DR: Diabetic retinopathy
OCT: Optical coherence tomography angiography
PDME: Persistent diabetic macular edema
VA: Visual acuity
